# Hidden Partners in Diversity: Acidobacteriota and Their Distribution in the Cape Floristic Region

**DOI:** 10.1002/mbo3.70192

**Published:** 2026-01-04

**Authors:** Janca Pieters, Karin Jacobs, Tersia Andrea Conradie

**Affiliations:** ^1^ Department of Microbiology Stellenbosch University, Stellenbosch Central Stellenbosch Western Cape South Africa

**Keywords:** *Acidobacteriota*, biodiversity hotspot, fynbos soils, soil abiotic factors, subdivisions

## Abstract

The Cape Floristic Region, a biodiversity hotspot in South Africa, is characterised by acidic, nutrient‐poor soils and distinctive fynbos vegetation. Despite the ecological importance and metabolic versatility of *Acidobacteriota*, their diversity and functional roles in fynbos soils remain poorly understood. This study investigated the diversity and abundance of *Acidobacteriota* in two nature reserves, Jonkershoek and Kogelberg, and the influence of soil abiotic factors and enzyme activities on their distribution and composition at the subdivision (SD) level. A total of 26 bulk soil samples were collected, and the V1–V9 regions of the 16S rRNA gene were sequenced using the Oxford Nanopore platform. The mean relative abundance of *Acidobacteriota* ranged from 1.5% to 36.25%. Subdivision 1 was the most dominant, with relative abundances of 66.96 ± 8.96% in Kogelberg Nature Reserve and 30.35 ± 0.15% in Jonkershoek Nature Reserve (*p* = 0.001). Other prevalent SDs included SDs 2, 3, and 5, with this study being the first to report the presence of SDs 22 and 17 in fynbos soils. Beta‐diversity analysis revealed distinct community compositions between the two reserves, driven by soil pH, moisture content, available phosphate, electrical conductivity, and enzyme activities (*p* = 0.001). Several positive and negative correlations between *Acidobacteriota* SDs and soil properties were also identified. Overall, this study highlights the high diversity of *Acidobacteriota* in fynbos soils and their close associations with soil abiotic properties, underscoring the need for cultivation‐based research to elucidate their ecological roles in these oligotrophic environments.

## Graphical abstract

*Acidobacteriota* diversity and abundance investigated in nutrient‐poor fynbos soils of the Cape Floristic Region. Nanopore 16S rRNA sequencing of 26 soil samples from Jonkershoek and Kogelberg identified dominant and rare subdivisions, revealed distinct community compositions, and demonstrated correlations between subdivisions and soil properties, including pH, moisture, phosphate, electrical conductivity, and enzyme activities.

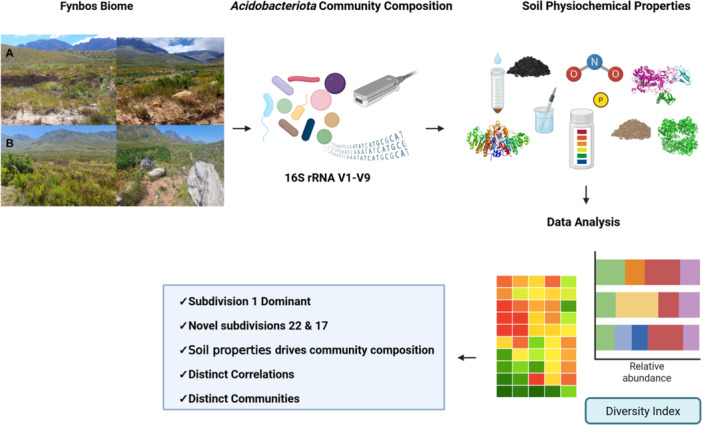

## 1 Introduction

The Cape Floristic Region (CFR), located in the Western and Southwestern regions of South Africa, is recognised as one of the world's biodiversity hotspots. Despite covering only 0.5% of South Africa's landmass, the CFR harbours over 20% of the continent's flora, including an estimated 9500 plant species, 68% of which are endemic (Critical Ecosystem Partnership Fund 2011). Central to the CFR is the Fynbos Biome, a fire‐prone shrubland characterised by a Mediterranean climate with hot, dry summers and cool, wet winters (Goldblatt and Manning 2002). Fynbos soils are naturally acidic and nutrient‐poor, particularly in nitrogen and phosphorus. The unique adaptation of fynbos plants to these oligotrophic conditions is facilitated, in part, by their symbiotic interactions with soil microbiota (Richards et al. 1997; Elliott et al. 2007; Lambers et al. 2011).

Despite extensive knowledge of fynbos plant diversity, the structure of bacterial communities inhabiting fynbos soils remains poorly understood, particularly regarding how abiotic and physicochemical soil properties shape these communities. Understanding the composition and functioning of bacterial assemblages in fynbos soils is crucial, as such insights provide essential baseline data for assessing the impacts of future ecological disturbances (Slabbert et al. 2014; Conradie and Jacobs 2021).

The phylum *Acidobacteriota*, first discovered in 1997, is currently divided into 26 phylogenetic subdivisions (SDs), with fewer than 100 successfully cultured and fully described species (Dedysh and Sinninghe Damsté 2018; Dedysh and Yilmaz 2018). *Acidobacteriota* is highly abundant and universally present in diverse environments, including soils, marine habitats, hot springs, and acid mine drainage (Barns et al. 1999; O'Connor‐Sánchez et al. 2014; Wegner and Liesack 2017). Within the Fynbos Biome, *Acidobacteriota* is particularly compelling due to its ability to thrive in acidic, nutrient‐poor soils, which are characteristic of this region. Known for tolerating oligotrophic conditions and for its capacity to degrade complex carbohydrates, this phylum plays a critical role in nutrient cycling in fynbos ecosystems, which are largely dependent on microbial activity (Sait et al. 2006; Fierer et al. 2012; Crits‐Christoph et al. 2018; Conradie and Jacobs 2021). Moreover, microbial diversity and composition are widely recognised as key drivers of ecological functioning. Therefore, it is essential to gain a deeper understanding of the factors that influence the distribution and composition of soil microbial communities.

Previous studies have shown *Acidobacteriota* as a dominant phylum in fynbos soils, with relative abundances ranging from 4% to 26%. However, a substantial knowledge gap remains, as approximately 40% of 16S rRNA gene sequences from these soils are unclassified (Slabbert et al. 2014; Postma et al. 2016; Keet et al. 2019; Conradie and Jacobs 2021). This highlights the potential of fynbos soils for novel discoveries within the phylum and underscores the need for further exploration of their ecological roles.

To better understand the diversity and ecological function of *Acidobacteriota* in fynbos ecosystems, this study compares the community composition of *Acidobacteriota* between two pristine fynbos nature reserves. The objectives are twofold: (1) to determine the relative abundance and diversity of *Acidobacteriota* in these soils, and (2) to assess the influence of environmental factors, including soil abiotic properties and, for the first time, soil enzymatic activities, on the composition of *Acidobacteriota*. This study aims to provide insights into how these bacteria interact with their environment in the unique fynbos biome.

## 2 Materials and Methods

### 2.1 Study Area and Sample Collection

**Figure 1:**
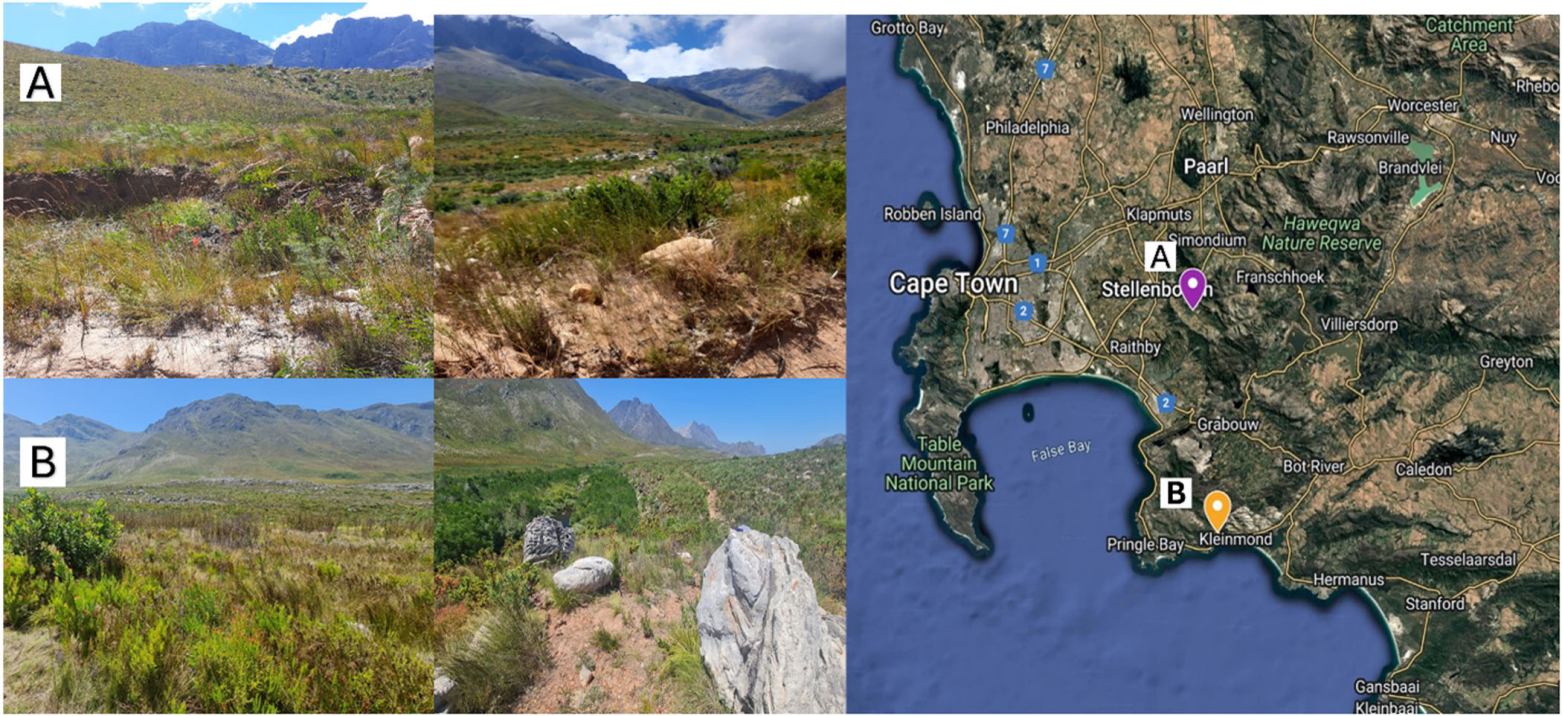
Visual representation of the different nature reserves sampled during this study. Jonkershoek Nature Reserve (A) and Kogelberg Nature Reserve (B).

The collection of soil samples was approved by the Western Cape Nature Conservation Board (permit: CN32‐87‐22071). Two sampling sites were selected based on their geographical location, fynbos types and diversity, namely Jonkershoek Nature Reserve (GPS 33° 58’ 7.0068” S; 18° 56’ 5.064” E) and Kogelberg Nature Reserve (GPS: 34° 19’ 55.416” S; 18° 59’ 15.972” E) (Figure 1). Jonkershoek Nature Reserve (JHR) is situated about 10 km southeast of Stellenbosch in the Western Cape. It's home to the Jonkershoek Mountains and a portion of the Jonkershoek Valley. Dominant fynbos present include Kogelberg Sandstone Fynbos, Cape Winelands Shale Fynbos and Boland Granite Fynbos. Soil in this environment is acidic and oligotrophic (Rebelo et al. 2006). The Kogelberg Nature Reserve (KGR) is widely recognised for its exceptional state of conservation. The reserve occupies a region minimally impacted by anthropogenic activities, resulting in an unparalleled level of fynbos biodiversity. Fynbos in this region includes Kogelberg Sandstone Fynbos and Western Coastal Shale Band, which are known for their acidic and nutrient‐poor soils (Rebelo et al. 2006).

A total of 26 bulk soil samples were collected between January and March in 2023. Briefly, at each site, random plots of 1 m × 1 m were identified (JHR *n* = 12; KGR *n* = 14) and three soil subsamples of approximately 150 g each were taken randomly within the first 10 cm of the soil surface. All samples were collected away from plants to avoid roots. Where present, the top layer of litter or organic material was removed before soil collection (Roesch et al. 2007; Keet et al. 2019). The three soil subsamples were combined and homogenised.

### 2.2 Physiochemical and Enzyme Profiles of Soil

Firstly, all tests were conducted in triplicate with appropriate standards and internal controls as required, and secondly, all absorbance readings were read on an iMark Microplate Absorbance Reader (Bio‐Rad, USA). Soil moisture, electrical conductivity (EC) and pH were measured as outlined in Anderson and Ingram (1994). Briefly, 10 g of soil was weighed into a sterile Falcon tube and incubated at 37°C for 7 days. The weight loss post‐incubation was recorded, and soil moisture was calculated (per gram of dry soil). The pH of the soil was measured in ddH_2_O solution (1:5) using an Ohaus Starter 2100 bench pH metre (Ohaus, Switzerland) and EC with Aqualytic AL15 electrochemistry metre (Lab Unlimited, Ireland).

A total of 0.1 g of soil was used for the remaining tests unless otherwise specified. To assess nitrogen mineralisation potential, ammonium concentrations in soil samples were measured on day 0 and day 7. Soil extracts were prepared using 0.5 M potassium sulphate. To each extract, Reagent N1 (0.085 g/mL sodium salicylate, 0.0625 g/mL sodium citrate, 0.0625 g/mL sodium tartrate, and 0.003 g/mL sodium nitroprusside) was added, followed by a 15‐min incubation to allow the reaction to proceed. Subsequently, Reagent N2 (0.04 g/mL sodium hydroxide and 2.5 mL sodium hypochlorite) was introduced to the mixture, and the samples were incubated for 1 h to develop colour. Absorbance was measured at 650 nm on days 0 and 7 (Anderson and Ingram 1994).

Nitrate levels were determined using a salicylic acid colourimetric method. Soil extracts were mixed with salicylic acid (5%) in sulphuric acid and left for 30 min. Sodium hydroxide (4 M) was then added, and the samples were incubated for 1 h to allow full‐colour development and absorbance measured at 410 nm. Available phosphorus was measured using Bray II solution, and phosphorus concentration in the extract was determined colourimetrically at 882 nm (Bray and Kurtz 1945). Organic carbon content was assessed using the Walkley‐Black method (Walkley 1947; Anderson and Ingram 1994), with absorbance readings taken at 600 nm. Active carbon, a labile fraction of soil organic carbon, was measured using the potassium permanganate oxidation method. All reagents were prepared as outlined in Weil et al. (2003). Samples were treated with 0.2 M potassium permanganate in a 1 M calcium chloride solution. The reaction was conducted in darkness for 10 min to ensure proper oxidation of the easily oxidisable organic matter. After allowing the soil to settle, 20 µL of the supernatant was transferred into a new tube containing 1.98 mL ddH_2_O. The absorbance of the diluted solution was measured at 550 nm.

Enzyme activities, including β‐glucosidase, urease, and alkaline and acid phosphatase, were measured following the protocol described by Dick et al. (1997). For each enzyme assay, 0.1 g of soil was used, with each sample tested in triplicate. β‐glucosidase activity was quantified using p‐nitrophenyl‐β‐d‐glucopyranoside (PNG) as substrate. The soil samples were incubated with the substrate and a buffer solution (THAM 12.1 g/L, Maleic acid 11.6 g/L, Boric acid 6.3 g/L, Citric acid 14.0 g/L, sodium hydroxide 19.52 g/L; pH 6.0) at 37°C for 1 h. After incubation, 0.5 M calcium chloride and THAM buffer (pH 12) were added, and absorbance measured (410 nm).

Urease activity was determined by incubating the soil samples in a 720 mM urea solution with borate buffer (pH 10) at 37°C for 2 h. After this incubation, a potassium hydroxide‐hydrochloric acid solution was added, and the mixture was incubated for an additional 30 min at room temperature and absorbance measured (660 nm).

Phosphatase activity was measured using p‐nitrophenyl phosphate (pNPP) as the substrate (0.025 M) for both acid and alkaline conditions. For acid phosphatase, soil samples were incubated with modified universal buffer (MUB) at pH 6.5, and the reaction was performed at 37°C for 1 h. The reaction was terminated with calcium chloride (0.5 M) and sodium hydroxide (0.5 M). The amount of p‐nitrophenol released was quantified spectrophotometrically at 410 nm, reflecting the enzyme's ability to release phosphate under acidic conditions. Alkaline phosphatase activity was similarly measured, with MUB adjusted to pH 11, representing phosphate release under alkaline conditions (Dick et al. 1997).

The fluorescein diacetate (FDA) hydrolysis assay was used to measure microbial activity in soil. Samples were incubated with FDA solution and a Tris buffer at pH 7.6. After 3 h of incubation at 37°C, the reaction was stopped with acetone, and the fluorescein produced measured at 490 nm (Green et al. 2006).

### 2.3 DNA Extraction and PCR Amplification

DNA of soil samples were extracted using the ZR *Quick*‐DNA Faecal/Soil Microbe Kits (Zymo Research, USA) according to the manufacturer's instructions with the following modifications to increase final DNA concentration; 0.1% (W/V) of Polyvinylpyrrolidone (PVP) was added to genomic lysis buffer and DNA Elution Buffer was heated at 65°C for 5 min on a heating block (Omega Scientific, USA). PCR amplification of successfully extracted genomic DNA was performed with primers specific to the V1‐V9 region of the 16S rRNA gene (Dos Santos et al. 2019). The total reaction volume (25 μL) contained 5 µL of 1x KAPA Taq Hotstart Buffer, 2 µL of 1.5 mM MgCl_2_, 0.5 μL dNTP mix, 075 μM forward [27 F (5’‐ACTCCTACGGGAGGCAGCAG‐3’)] and reverse primers [1492 R (5’‐GGTTACCTTGTTACGAGTT‐3’)], 0.2 µL of 0.5 U KAPA Taq HotStart DNA Polymerase, and 2 μL of template DNA. Products were amplified using the 2720 Thermal Cycler (Applied Biosystems, USA) with following settings: Initial denaturing at 95°C for 5 min, followed by 35 cycles of 95°C for 30 s, 58°C for 30 s and 72°C for 1 min. A final extension was completed at 72°C for 1 min, and the PCR samples were held at 4°C. Successful amplification was visualised on a 1% agarose gel stained with ethidium bromide, and final DNA concentration measured with BioDrop µLite (BioDrop, UK).

### 2.4 Barcoding and Library Preparation

Following amplification, DNA barcoding and library preparation were performed with the Native Barcoding Kit 96 V14 (SQK‐NBD114.96, Oxford Nanopore Technologies (ONT), UK) according to the manufacturer's instructions. Briefly, for end preparation and dA ‐tailing the NEBNext Ultra II module (ONT, UK) was used with 11.5 µL of amplified DNA (final concentration of 200 fmol). The reaction was completed by incubating at 20°C for 5 min and 65°C for 5 min on a 2720 Thermal Cycler (Applied Biosystems, USA). These products were then assigned a unique barcode and pooled. Adaptors were ligated and cleaned with the NEBNext Quick Ligation Module (ONT, UK) per manufacturer's instructions. The long fragment buffer was used to select the correct fragment size (1500 to 1900bp range). The final library concentration was determined with BioDrop µlite (BioDrop, UK) and was made to 12 µL (50 fmol) for loading. The prepared sequencing library and Flow Cell Priming Kit (ONT, UK) were loaded onto an R10.4.1 flow cell (ONT, UK). The flow cell was run using the MinION Mk1B sequencing device through the MinKNOW software (Version 24.02.6). Basecalling was performed with Dorado (Version 7.2.0) integrated into the MinKNOW software (Version 24.02.6) with the super accurate base calling model. Barcode trimming was enabled for both ends and middle.

### 2.5 Amplicon Sequencing Data Processing

The quality of the reads was assessed and visualised using Nanoplot (Version 1.42.0; de Coster et al. 2018). Firstly, all primer sequences, sequences with ambiguous bases and homopolymers longer than 8 bp were removed. Secondly, sequences were filtered based on size (1000bp‐1900bp) and quality (phred score = 98%). Following read quality assessment, data were analysed using MOTHUR (Version 1.48.0; Schloss et al. 2009; Winand et al. 2019; Akaçin et al. 2023). All chimeric sequences were identified and removed using VSEARCH (Version 2.16.0; Rognes et al. 2016). After quality filtering and trimming, the remaining sequences were clustered into OTUs (97%) and classified against two databases, SILVA Version 1.32 (http://www.arb-silva.de/) reference database and EzBioCloud (https://www.ezbiocloud.net/) with a cut‐off value of 80 (Quast et al. 2012; Yoon et al. 2017; Chalita et al. 2024). All sequences classified as unknown, eukaryotes, mitochondria, or chloroplasts were removed before further analysis. The raw sequence data were submitted to GENBANK as FASTQ files with Bioproject number **PRJNA1207934**.

### 2.6 Data Analysis

All statistical analyses were conducted in R (Version 2024.04.2; R Core Team 2024). Data normality was assessed using the Shapiro‐Wilk Test with the Hmisc package (Version 5.1.3; Harrell 2024), and downstream analyses were adjusted accordingly. Alpha‐diversity, relative abundance and beta‐diversity indices were determined using the Microeco package (Version 1.8.0; Liu et al. 2021) and visualised with ggplot2 (Version 3.5.; Wickham and Wickham 2016). Mann‐Whitney U tests were performed to compare Shannon and Simpson indices between nature reserves. Permutational Multivariate Analysis of Variance (PERMANOVA) was performed to determine the significance of beta‐diversity differences between the nature reserves with the Vegan package (Version 2.6.8; Oksanen et al. 2024) and Adnois2 function (Oksanen et al. 2024). Spearman's rank correlation tests were conducted between soil abiotic properties and *Acidobacteriota* SDs using Hmisc, and the results were visualised with pheatmap (Version 5.1.3; Harrell 2024. Correlations between *Acidobacteriota* SDs and the two nature reserves were evaluated using nonmetric multidimensional scaling (NMDS) ordinations with 9999 permutations in the Vegan package (Version 2.6.8; Oksanen et al. 2024). The NMDS plots were based on Bray‐Curtis distance matrices. For all statistical tests, a *p* < 0.05 was considered significant.

## 3 Results

### 3.1 Soil Physiochemical Factors and Enzyme Activity

The soil abiotic properties and enzyme activities for each site (mean ± standard deviation), together with the significant *p*‐values, are summarised in Table 1. A significant difference was observed for pH, EC, moisture content, nitrate, nitrogen mineralisation, alkaline phosphatase, urease, and available phosphate. The mean soil pH values ranged from 5.28 ± 0.27 (JHR) to 6.51 ± 0.03 (KGR), reflecting the generally acidic conditions characteristic of fynbos soils. The amount of nitrate was significantly higher in KGR 10.67 ± 8.16() compared to JHR (0.52 ± 0.28). The same trend was observed for nitrogen mineralisation (JHR = 0.07 ± 0.16; KGR = 0.6 ± 0.49). Acid and alkaline phosphatase activity was higher in KGR compared to JHR.

**Table 1:** Soil abiotic properties and enzyme activities for each nature reserve. Values are means ± standard deviation (Jonkershoek Nature Reserve *n* = 12, Kogelberg Nature Reserve *n* = 14).

Property	Jonkershoek	Kogelberg	Significance
EC	66.26 ± 13.37	41.34 ± 34.29	***
pH	5.28 ± 0.27	5.71 ± 0.61	**
Moisture content (%)	0.98 ± 0.03	0.97 ± 0.02	*
Acid phosphatase (µmol/g/h)	439.04 ± 238.65	655.18 ± 318.51	NS
Alkaline phosphatase (µmol/g/h)	368.01 ± 208.9	639.29 ± 141.66	**
β‐Glucosidase (µmol/g/h)	1089.78 ± 175.51	980.34 ± 46.77	NS
Urease (µmol/g/h)	0.35 ± 1.11	1.75 ± 0.76	***
Nitrate (mg/kg)	0.52 ± 0.28	10.67 ± 8.16	***
Nitrogen mineralisation (mg/kg)	0.07 ± 0.16	0.6 ± 0.49	***
Available phosphate (mg/kg)	1.13 ± 0.45	0.63 ± 0.53	*
Organic carbon (%)	0.25 ± 0.1	0.58 ± 0.48	NS
Active carbon (mg/kg)	609.26 ± 186.74	686.68 ± 113.12	NS
Microbial activity (µmol/g/h)	277.39 ± 156.04	337.38 ± 178.7	NS

A significant difference is observed at: *p < 0.05; **p < 0.01; ***p < 0.001; NS, not significant.

### 3.2 *Acidobacteriota* Diversity in Nature Reserves

#### 3.2.1 Abundance and Alpha‐Diversity

After quality filtering and removal of chimeras, a combined total of 77,074,902 partial 16S rRNA gene sequences with a mean amplicon length between 1200 and 1800 bp were obtained. A total of 104,426 ( ~ 35.14%) were classified as *Acidobacteriota*‐affiliated reads. The relative abundance of *Acidobacteriota* ranged from 1.5% to 36.25% and was the most dominant phylum at both sites. There was no significant difference in the mean relative abundance of overall *Acidobacteriota* between JHR and KGR. Other major taxa identified across all nature reserve samples included the phyla *Pseudomonadota*, *Actinomycetota*, *Planctomycetota*, and *Bacillota*. The mean Shannon and Simpson diversity indices for JHR were 2.79 ± 0.76 and 0.901 ± 0.33, respectively. Kogelberg Nature Reserve had a Shannon diversity index of 2.82 ± 0.23 and 0.902 ± 0.32 for Simpson. The difference observed for Shannon diversity index between the two sites was significant (Table 2).

**Table 2:** The overall Shannon and Simpson indices of Jonkershoek and Kogelberg nature reserves.

Test	Jonkershoek	Kogelberg	Significance
Shannon	3.92 ± 0.44	4.29 ± 0.23	**
Simpson	0.964 ± 0.01	0.973 ± 0.973	***

A significant difference is observed at: *p < 0.05; **p < 0.01; ***p < 0.001.

**Figure 2:**
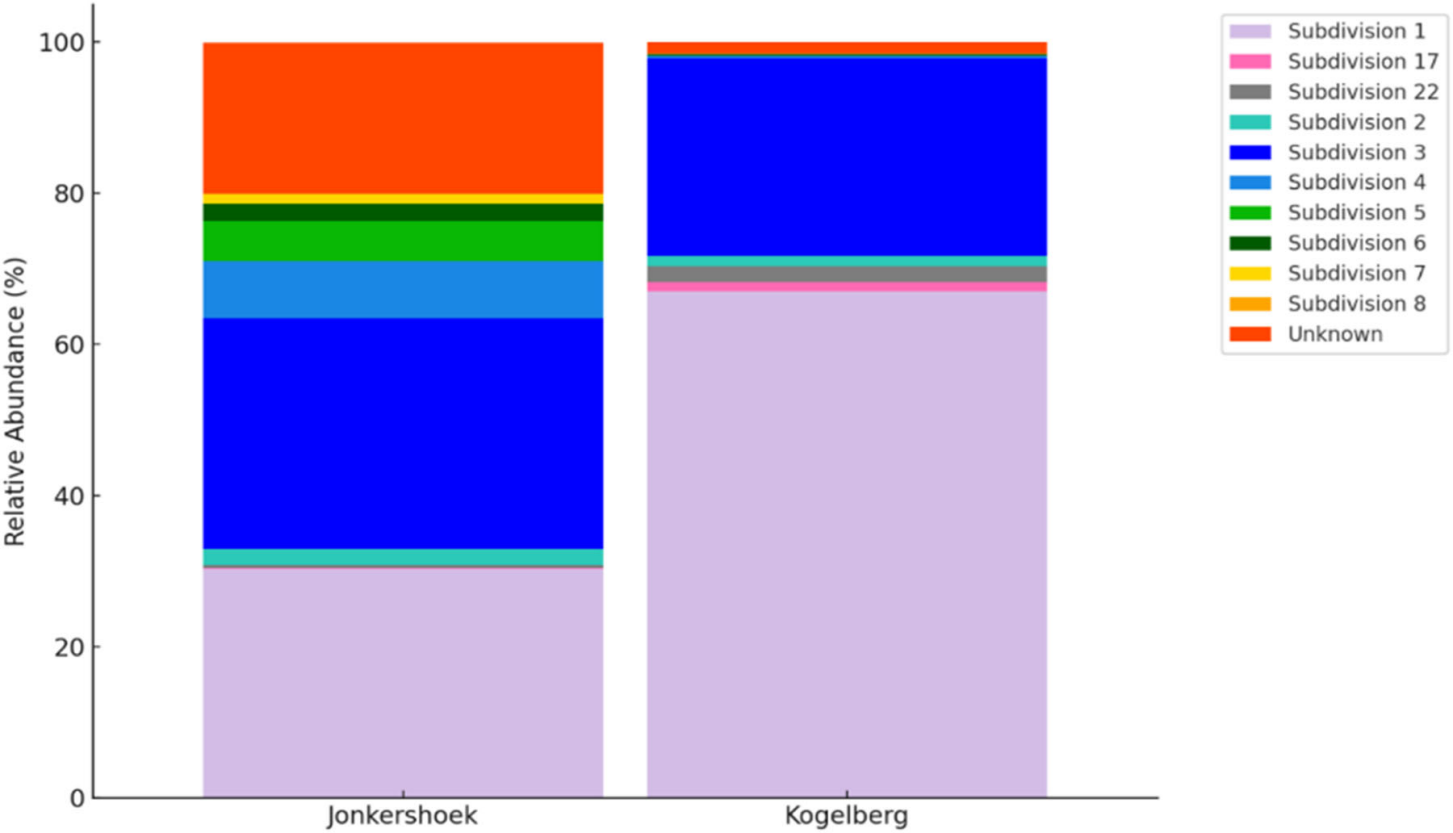
The relative abundance (%) of the different *Acidobacteriota* subdivisions observed at both Jonkershoek Nature Reserve and Kogelberg Nature Reserve.

*Acidobacteriota* OTUs were classified into 23 phylotypes, representing members from various SDs (Table 3). The major *Acidobacteriota* community composition and relative abundance at the two sites are depicted in Figure 2. The majority of the OTUs identified belong to SDs 1, 3, 2 and 4 (Figure 2, Table 3). In the JHR samples, the Acidobacteriota community was primarily composed of SDs 3 and 1, which accounted for 30.58% and 30.35%, respectively, followed by SD 4, representing 7.59%. In contrast, SD 1 represented more than half of the Acidobacteriota community in Kogelberg, followed by SDs 3 and 2 (Table 3). Certain SDs were exclusive to the two sites: SD 7 to KGR and SD 8 to JHR. A significant difference was observed in the relative abundance of SDs 4 and 6, which were more than twice as abundant in JHR compared to KGR. Subdivisions with a relative abundance of less than 1% in JHR included 17 and 22, in contrast, SDs 4, 5, 6 and 8 displayed a relative abundance of less than 1% KGR. Known genera from SD 1 included members from *Acidipila*, *Occallatibacte*r, *Candidatus* Korobacter, *Granulicella*, *Acidicapsa*, and *Bryocella*. Members of SD 3 included *Candidatus* Solibacter, *Bryobacter* and *Paludibaculum*. Subdivision 4 members present were *Pyrinomonas, Blastocatella* and *Stenotrophobacter*. A total of 18.33% of OTUs accounted for Unknown SDs (unclassified) in JHR and only 1.26% in KGR.

**Table 3:** The relative abundance (%) of *Acidobacteriota* subdivisions observed in Jonkershoek nature reserve and Kogelberg nature reserve.

Subdivision	Jonkershoek	Kogelberg	Significance
Subdivision 1	30.35 ± 0.15	66.96 ± 8.96	***
Subdivision 17	0.07 ± 0.16	1.27 ± 1.44	**
Subdivision 22	0.44 ± 0.92	2.12 ± 6.41	NS
Subdivision 2	2.12 ± 2.34	1.31 ± 8.30	***
Subdivision 3	30.58 ± 4.30	26.20 ± 3.85	NS
Subdivision 4	7.59 ± 18.13	0.29 ± 0.55	**
Subdivision 5	5.18 ± 23.04	0.03 ± 0.04	**
Subdivision 6	2.37 ± 6.30	0.21 ± 0.50	**
Subdivision 7	1.29 ± 2.61	0	**
Subdivision 8	0	0.06 ± 0.15	NS
Unknown	19.00 ± 0.02	1.54 ± 0.43	***

A significant difference is observed at: **p < 0.01; ***p < 0.001; NS, Not Significant

#### 3.2.2 Beta‐Diversity

**Figure 3:**
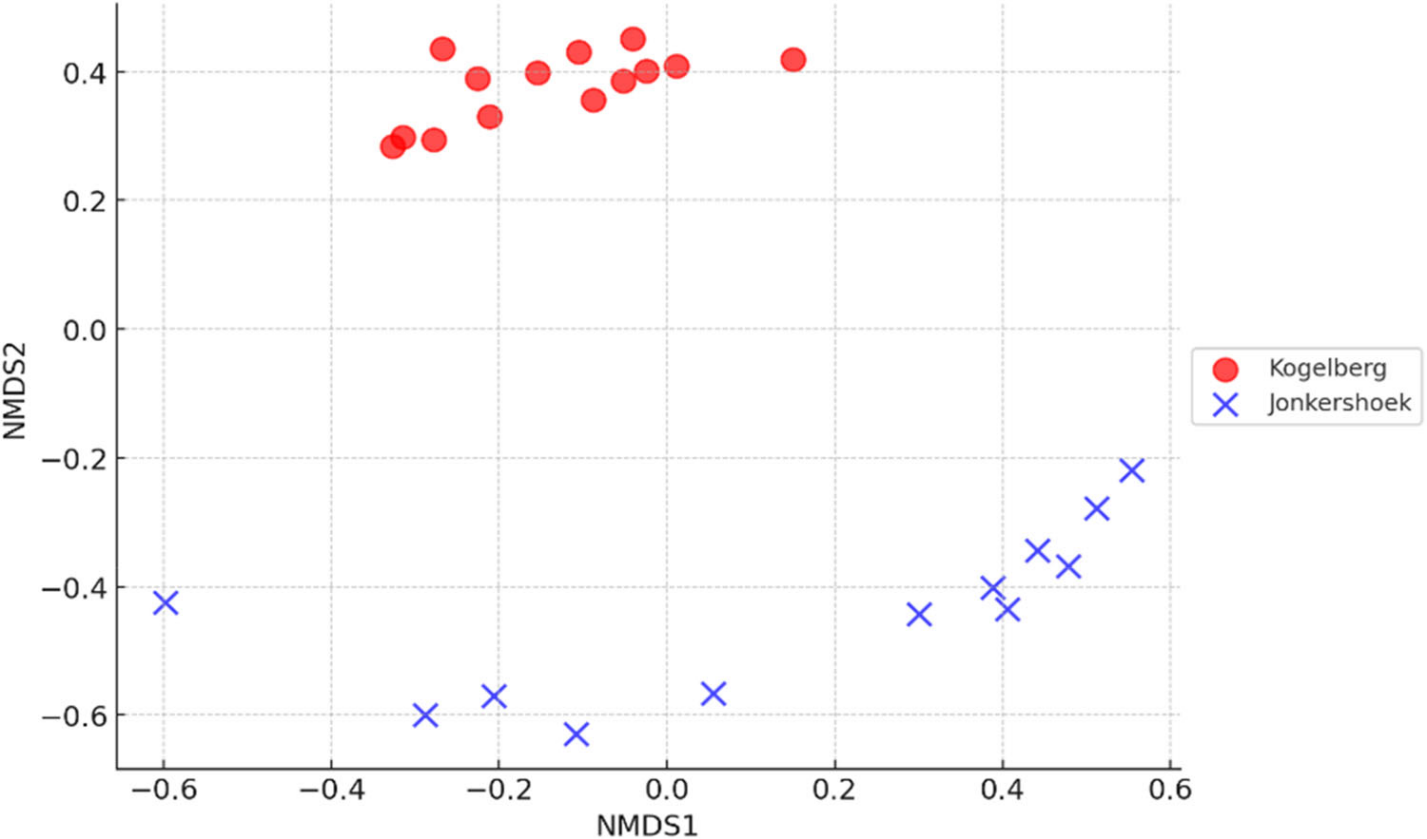
A nonmetric multidimensional scaling ordination plot (NMDS), based on the Bray‐Curtis distance matrix, representing the *Acidobacteriota* community compositions in various samples examined from Jonkershoek Nature Reserve and Kogelberg Nature Reserve.

**Figure 4:**
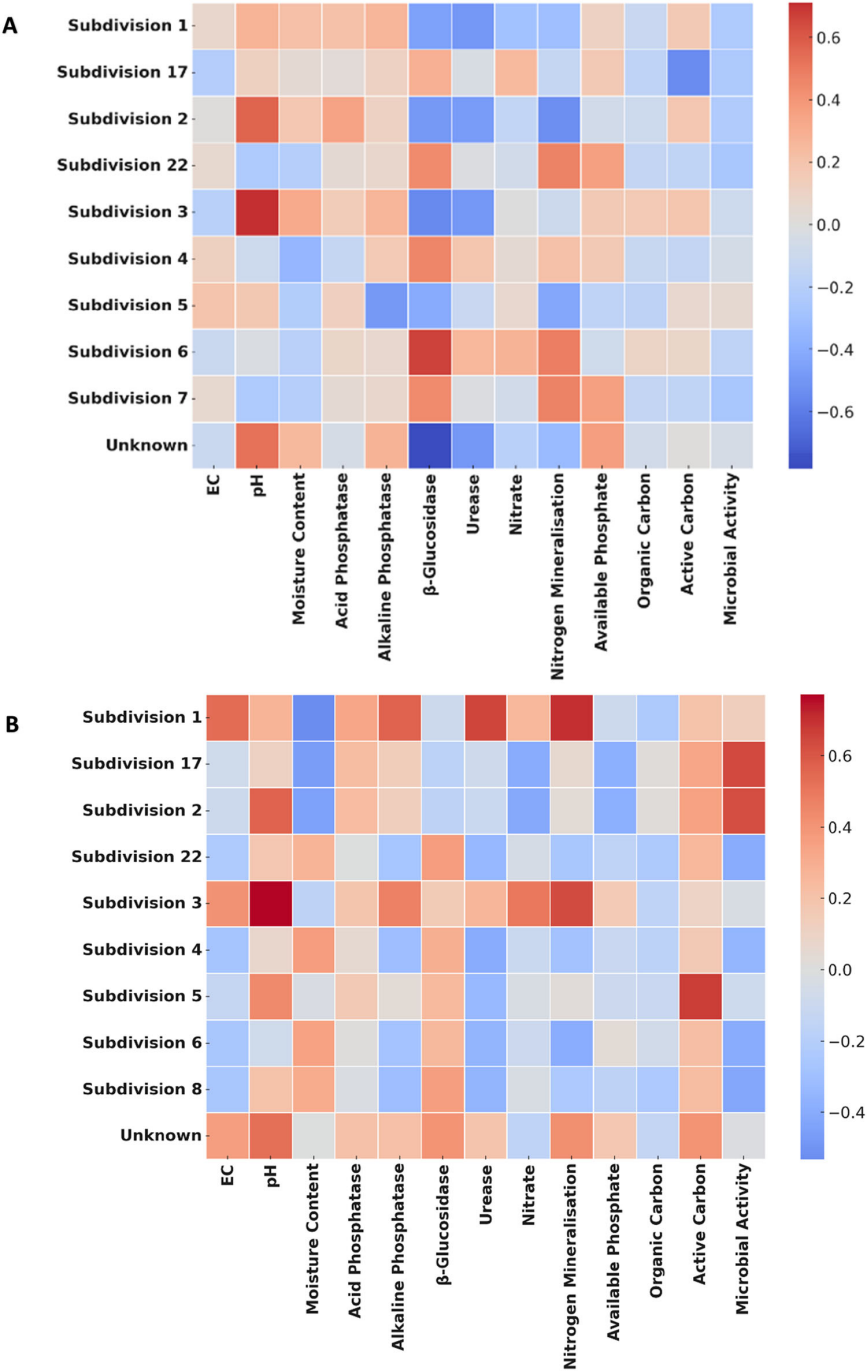
Spearman's rank correlations for the different *Acidobacteriota* subdivisions, soil properties and enzyme activities at Jonkershoek Nature Reserve (A) and Kogelberg Nature Reserve (B). Colour of the square indicates type of correlation (shades of dark red–positive and blue negative).

NMDS revealed distinct Acidobacteriota community compositions among the sampling sites (Figure 3). The observed separation between JHR and KGR differed significantly (*p* = 0.001). Soil abiotic properties responsible for this separation included soil pH and EC, soil enzyme activities of alkaline phosphatases and β‐glucosidase (Figure 4 **).** Correlations between specific *Acidobacteriota* 0 s, soil abiotic properties and enzyme activities were examined to identify significant positive and negative correlations. In KGR, SD 1 displayed positive correlations with EC (*p* = 0.04; ρ = 0.53), alkaline phosphatases (*p* = 0.03; ρ = 0.56), nitrogen mineralisation (*p* = 0.005; *ρ* = 0.699) and urease (*p* = 0.007; *ρ* = 0.652). Subdivision 3 displayed a positive correlation with nitrogen mineralisation (*p* = 0.001; ρ = 0.630) and urease (*p* = 0.03; *ρ* = 0.564). Both SD 2 (*p* = 0.005; *ρ* = −0.566) and SD 3 (*p* = 0.001; *ρ* = 0.711) displayed a significant correlation with pH.

## 4 Discussion

### 4.1 Overall Relative Abundance and Different Compositions of Subdivisions

This study confirms that fynbos soils harbour diverse members of both classified and unknown *Acidobacteriota*. Overall, the major SDs identified during this study were SDs 1, 3, 4, 5 and 6 (Figure 2, Table 3). Previous research on microbial community diversity in fynbos soils has reported *Acidobacteriota* relative abundances ranging from 4% to 26% (Keet et al. 2019; Conradie and Jacobs 2021). Consistent with previous studies, a similar trend was observed in this study, with *Acidobacteriota* accounting for 36.25% of the total bacterial community. The dominant members were SD 1 (class *Terriglobia*, order *Terriglobales*) and SD 3 (class *Terriglobia*, order *Bryobacterales*. Both of which are consistently abundant in diverse soil environments (Kielak et al. 2016; Dedysh and Sinninghe Damsté 2018; Eichorst et al. 2018).

The Shannon and Simpson indices were higher in KGR than in JHR, indicating a greater species diversity that is more evenly distributed (Wang et al. 2022). The beta‐diversity was also significantly different between the two sites. JHR exhibited greater variability in species composition across samples, suggesting greater spatial heterogeneity, while the *Acidobacteriota* community in KGR had less variation between samples. Furthermore, the difference in beta‐diversity is driven by variations in soil abiotic properties, including significant differences in pH, nitrate and available phosphate, as well as differences in enzyme activities of urease and alkaline phosphatases. The influence of abiotic factors on bacterial community shifts is well established (Islam et al. 2020; Walters and Martiny 2020).

Subdivision 1 is the most well‐studied and represented SD of the phylum *Acidobacteriota* and has been identified as one of the most dominant SDs in soil and other environmental settings (Huber et al. 2022). In this study, members of SD 1 included genera such as *Acidipila*, *Occallatibacter*, *Candidatus* Korobacter, *Granulicella*, *Acidicapsa*, and *Bryocella*. These genera are present in fynbos soils due to their physiological characteristics, which enable them to thrive in this environment. These characteristics include their ability to utilise various sugars and polysaccharides, different electron acceptors, and tolerate a wide range of pH, as documented in several studies (Koch et al. 2008; Okamura et al. 2011; Foesel et al. 2016; Jiang et al. 2016; Xia et al. 2017; Belova et al. 2018). Soil pH is widely recognised as a key determinant of soil microbial structure and composition, a trend that holds for SD 1 (Chu et al. 2010; Fernández‐Calviño and Bååth 2010; Rousk et al. 2010; Wan et al. 2020). Extreme pH conditions, whether high or low, can damage microbial cells, resulting in an increase in nutrient availability. This, in turn, creates opportunities for bacterial communities, such as *Acidobacteriota*, to thrive. Additionally, soil pH indirectly affects bacterial communities by altering elemental solubility, nutrient cycling, and substrate availability, which significantly influence bacterial growth and activity (Kemmitt et al. 2006). Previous studies have shown that SD 1 and 3 generally prefer lower pH environments (Conradie and Jacobs 2021; Gonçalves et al. 2024). However, in KGR, SD 1 abundance increased slightly with rising pH, suggesting taxon‐specific responses within the same SD. This demonstrates that pH effects on bacterial communities can vary even among closely related taxa (Zhou et al. 2024). A similar pattern was observed in JHR, where SD 3 exhibited a positive correlation with pH, indicating its ability to tolerate a broader pH range. This reinforces the taxon‐specific adaptability within *Acidobacteriota*. In contrast, SD 2 showed a negative correlation with pH, aligning with findings by Ivanova et al. (2020). However, other studies (Conradie and Jacobs 2021; Tong et al. 2024) reported a positive correlation, suggesting that SD 2 demonstrates remarkable versatility in adapting to different pH conditions. This variability underscores the limited understanding of the ecological roles and adaptability of this phylum.

Genes involved in phosphorus metabolism have been identified in SD 1 members, suggesting a possible role in phosphorus solubilisation in the environment (Ma et al. 2024). Interestingly, in this study, a positive correlation was observed in alkaline phosphatase activity in KGR, suggesting that these organisms may solubilise phosphorus in environments with slightly higher pH, such as those observed in KGR, despite their usual preference for acidic conditions. This further highlights the ability of members in the same SD to survive in different environments, as mentioned above. These insights may inform future cultivation strategies tailored to a specific environment, as different members of *Acidobacteriota* might employ different survival strategies. Urease activity plays a crucial role in nitrogen‐limited soils. This activity is particularly important in soils where nitrogen is a limiting factor for plant growth, as it enhances soil fertility. Members of SD 1 catalyse the hydrolysis of urea into ammonia and carbon dioxide, making nitrogen available for plant and microbial use (Gonçalves et al. 2024). Song et al. (2024) also found a positive correlation between urease activity and SD 1. However, the potential contribution of other microorganisms or fungi to the enzyme activity should not be disregarded.

C*andidatus* Solibacter and *Bryobacter* from SD 3 can use nitrate as an electron acceptor, which might explain and clarify the positive correlation observed with nitrogen mineralisation in this study (Deng et al. 2018). Moreover, studies have linked these genera to key soil functions, such as organic matter decomposition and nitrogen metabolism, indicating their important contribution to maintaining soil health under varying nitrogen conditions (Jara‐Servin et al. 2023). It is important to note that these findings indicate a correlation rather than a direct functional role of SD 1 and SD 3 in the nitrogen cycle, as more functional studies are required. Additionally, the interactions between this phylum and other bacterial species in the environment should not be ignored.

Subdivision 4 includes members such as the family *Pyrinomonadaceae*, the uncultivated clade RB41, and the genera *Stenotrophobacter* and *Pyrinomonas*, which are known to thrive in warmer climates (Foesel et al. 2013). Cultivated members of this SD have demonstrated optimal growth at pH levels ranging from 5.07 to 7.0 (Wüst et al. 2016). As the fynbos biome is characterised by warm summers and lower pH levels, their presence here is expected. Phenotypic, chemotaxonomic, and phylogenetic analyses suggest that SD 4's adaptability to thrive in diverse environments is due to its metabolic versatility (Parsley et al. 2011; Kielak et al. 2016; Coluccia and Besaury 2023). Members of this SD have been shown to utilise chitin as a carbon source (Foesel et al. 2013; Huber et al. 2014), which has significant implications for low‐nutrient soils. In these environments, where bioavailable carbon and nitrogen are often scarce, chitin degradation provides a crucial alternative nutrient source, facilitating the release of ammonia, ammonium and nitrate, which are essential for microbial and plant growth (Beier and Bertilsson 2013). The ability to degrade complex organic matter such as chitin allows these bacteria to persist in oligotrophic soils, reducing competition for simpler carbon sources and enhancing microbial survival under nutrient‐limited conditions (Goldfarb et al. 2011). Furthermore, microbial chitin degradation contributes to soil organic matter formation and carbon sequestration, promoting long‐term soil stability, water retention, and nutrient availability in ecosystems such as fynbos, Arctic tundra, and acidic forest soils (Rumpel and Kögel‐Knabner 2011). Given these ecological roles, SD 4 likely plays a key role in nutrient cycling, microbial community structuring, and soil resilience in nutrient‐poor environments.

The relative abundance of SD 5 and 6 differed significantly between KGR and JHR (Table 3). Currently, SD 6 contains two taxonomically described members: *Vicinamibacter silvestris* and *Luteitalea pratensis* that belong to the family *Vicinamibacteraceae*. Members of this family are aerobic, neutrophilic, psychrotolerant to mesophilic chemoheterotrophs that grow on sugars or complex proteinaceous compounds and can tolerate a broad range of pH values (Huang et al. 2016; Dedysh and Yilmaz 2018). Subdivision 5 is currently placed under the class *Terriglobales*; however, no additional information is available. The preference for JHR compared to KGR might be due to the variability in nutrient availability and soil properties that differ between the two sites, as the effect of soil abiotic factors on microbial composition is well established (Fierer and Jackson 2006; Wang et al. 2022). This holds true for SDs unique to specific sites, such as SD 7 in KGR and SD 8 in JHR. These findings indicate that microbial community composition is largely determined by site‐specific environmental conditions. Site‐specific soil properties, including pH, nutrient availability, EC, and moisture content, create distinct ecological niches that selectively favour particular taxa.

Subdivisions 22 and 17 were identified in this study, marking this the first report of their presence in the fynbos environment, albeit at a very low abundance (Table 3). Subdivision 22 forms part of the class *Holophagae*, with the only cultivated representative *Candidatus* Polarisedimenticola, which was isolated from marine sediments (Dedysh and Yilmaz 2018; Flieder et al. 2021). Subdivision 17 has no cultured representatives and is tentatively classified within the same class as SD 6, *Vicinamibacteria* (Dedysh and Yilmaz 2018). The detection of these SDs in this study may be attributed to ONT sequencing, which enables the identification of previously undetected or low‐abundance taxa. The increased resolution and species detection power of ONT sequencing have been widely documented, particularly in improving taxonomic classification and enhancing the recovery of rare microbial lineages (Stevens et al. 2023; Szoboszlay et al. 2023). This suggests that advanced sequencing technologies may provide a more comprehensive view of *Acidobacteriota* diversity than traditional sequencing approaches.

## 5 Conclusion

The phylum *Acidobacteriota* is a unique and diverse group of bacteria that has garnered significant attention over the past decade due to its presence in a wide range of environments. Their frequent detection in oligotrophic and acidic soils underscores their ecological importance. This study provides valuable insights into the diversity and distribution of *Acidobacteriota* in fynbos soils and their correlations with key soil properties and enzymatic activities.

The relative abundance of *Acidobacteriota* SDs varied significantly between Jonkershoek Nature Reserve and Kogelberg Nature Reserve, with major SDs identified as SDs 1, 3, 4, and 5. A key finding of this study is the substantial variation in Acidobacteriota community composition between the two sites, driven by differences in abiotic factors, including soil pH and enzymatic activities (urease and alkaline phosphatase). These findings highlight the adaptability of *Acidobacteriota* to diverse environmental conditions, demonstrating that members of the same taxa may have distinct ecological preferences. For example, SDs 1, 3, and 2 were shown to tolerate and thrive in environments with differing soil pH levels, indicating functional and ecological versatility within these SDs.

The study also documented higher relative abundances of SD 4 and SD 5 compared to previous reports, suggesting site‐specific adaptations or shifts in community composition. Moreover, previously uncharacterised SDs, such as SD 17 and SD 22, were identified in these fynbos soils, highlighting the potential of advanced sequencing technologies, such as ONT, in uncovering hidden microbial diversity. However, a considerable proportion of sequences remained unclassified, emphasising the limitations of current databases in fully capturing the diversity of *Acidobacteriota*.

Despite these important findings, the study emphasises gaps in our understanding of *Acidobacteriota* and their ecological roles. The lack of cultured representatives for many SDs constrains our ability to draw definitive conclusions about their specific functions and contributions to soil ecosystems. This limitation underscores the need for future research focused on the isolation, cultivation, and genomic characterisation of *Acidobacteriota*. Such efforts will provide deeper insights into the ecological significance and functional potential of this enigmatic phylum, particularly in unique environments like fynbos soils.

## Author Contributions

**Janca Pieters:** conceptualization, formal analysis, investigation, methodology, data curation, visualization, writing – original draft. **Karin Jacobs:** project administration, resources, supervision, writing – review and editing. **Tersia Andrea Conradie:** conceptualization, project administration, supervision, writing – review and editing.

## Ethics Statement

The authors have nothing to report.

## Conflicts of Interest

The authors declare no conflicts of interest.

## Data Availability

The data that support the findings of this study are openly available in Genbank at https://www.ncbi.nlm.nih.gov/search/all/?term=PRJNA1207934, reference number PRJNA1207934.
